# Integrated Omics Approaches Revealed the Osmotic Stress-Responsive Genes and Microbiota in Gill of Marine Medaka

**DOI:** 10.1128/msystems.00047-22

**Published:** 2022-03-14

**Authors:** Keng Po Lai, Peng Zhu, Delbert Almerick T. Boncan, Lu Yang, Cherry Chi Tim Leung, Jeff Cheuk Hin Ho, Xiao Lin, Ting Fung Chan, Richard Yuen Chong Kong, William Ka Fai Tse

**Affiliations:** a Laboratory of Environmental Pollution and Integrative Omics, Guilin Medical University, Guilin, China; b Guangxi Key Laboratory of Beibu Gulf Marine Biodiversity Conservation, Beibu Gulf University, Qinzhou, China; c Department of Chemistry, City University of Hong Kong, Hong Kong SAR, China; d State Key Laboratory of Marine Pollution, City University of Hong Kong, Hong Kong SAR, China; e School of Life Sciences, State Key Laboratory of Agrobiotechnology, The Chinese University of Hong Kong, Hong Kong SAR, China; f Department of Psychiatry, Icahn School of Medicine at Mount Sinai, New York, New York, USA; g Research Centre for the Oceans and Human Health, City University of Hong Kong Shenzhen Research Institute, Shenzhen, China; h Laboratory of Developmental Disorders and Toxicology, Center for Promotion of International Education and Research, Faculty of Agriculture, Kyushu University, Fukuoka, Japan; University of Hawaii at Manoa

**Keywords:** gill, medaka, hypotonic stress, osmosensing, osmotic stress, RNA sequencingmetagenomics, osmoregulation

## Abstract

Aquatic fishes face osmotic stress continuously, and the gill is the first tissue that senses and responds to the external osmotic challenges. However, the understandings of how the gill microbiota could respond to osmotic stress and their potential host-bacterium relationships are limited. The objectives of the current study are to identify the hypotonic responsive genes in the gill cells and profile the gill microbiota communities after fresh water transfer experiment via transcriptome sequencing and 16S rRNA gene sequencing. Transcriptome sequencing identified 1,034 differentially expressed genes (DEGs), such as aquaporin and sodium potassium chloride cotransporter, after the fresh water transfer. Gene Ontology (GO) and Kyoto Encyclopedia of Genes and Genomes (KEGG) analysis further highlighted the steroid biosynthesis and glycosaminoglycan biosynthesis pathways in the gill. Moreover, the 16S rRNA gene sequencing identified *Vibrio* as the dominant bacterium in the seawater, which changed to Pseudomonas and *Cetobacterium* after the fresh water transfer. The alpha diversity analysis suggested that the gill bacterial diversity was lower in the fresh water transferred group. The KEGG and MetaCyc analysis further predicted the alteration of the glycosaminoglycan and chitin metabolisms in the gill bacteria. Collectively, the common glycosaminoglycan and chitin pathways in both the gill cells and gill microbiota suggest the host-bacterium interaction in gill facilitates the fresh water acclimation.

**IMPORTANCE** This is the first study using the transcriptome and 16S rRNA gene sequencing to report the hypotonic responsive genes in gill cells and the compositions of gill microbiota in marine medaka. The overlapped glycosaminoglycan- and chitin-related pathways suggest host-bacterium interaction in fish gill during osmotic stress.

## INTRODUCTION

Ion and water osmoregulation is critical for the maintenance of tissue and cellular functions. It is important to define cell shape, intracellular osmolality, and various cellular functions (1). Fishes have an osmoregulatory mechanism to regulate fluid and ion homeostasis to maintain a constant body osmolality during osmotic stress. The gill is the major tissue for the osmoregulatory processes. Early studies using electron microscopic analysis reported remodeling of gill cells in different salinities (2, 3). In previous decades, general characterizations of selected ion transporters and hormonal receptors, such as localization, and mRNA/protein expression levels were studied. Recent omics approaches have gained insights on the genomic or proteomic responses in different fish models (4–8). Medaka (*Oryzias* spp.) inhabit diverse osmotic environments worldwide (9). Due to their salinity adaptability, medaka species have been utilized in research to understand the osmoregulatory mechanisms involved in fresh water or seawater acclimation (10, 11). Marine medaka (*Oryzias melastigma*) is native to coastal waters. It can be found in mangroves and has the ability to adapt and spawn in freshwater (12). In the last few years, marine medaka has been used to study the environmental marine pollution (13, 14) due to its seawater habitat and well-known genome (15, 16). However, most of the salinity works in medaka species were performed in the Japanese medaka (*Oryzias latipes*) (10, 17–20), which lives in fresh water. The current study applied RNA sequencing to provide an overview of the hypotonic osmoregulatory mechanism in gill of marine medaka.

On the other hand, our understandings of the osmoregulatory mechanism have mainly focused on the fish itself. The effects of osmotic stress on gill bacteria are not known. Recent studies have shown the changes of taxonomic microbial compositions in gut across environmental salinity (21). The gut bacteria are suggested to play roles in nutrient absorption and immune response for host survival (22, 23). Regarding the marine medaka, a current report has demonstrated the shift of the gut bacterial communities after the seawater-to-fresh-water transfer experiment (24). Since the gill is continuously exposed to the external media, the gill bacteria must develop a mechanism to compensate for the osmotic stress. To summarize, this is the first study to integrate the transcriptomics and metagenomics approaches to understand the responses of gill and the microbiota under the hypotonic stress.

## RESULTS

This study used marine medaka (*Oryzias melastigma*) to study the genome-wide changes of gene expression in gill and gill microbiota communities upon hypotonic stress. For the first part of the study, we conducted transcriptomic analysis to identify differentially expressed genes (DEGs) and the genome-wide molecular regulatory networks after fresh water transfer in marine medaka.

### General sequencing information of the transcriptome.

**FIG 1:**
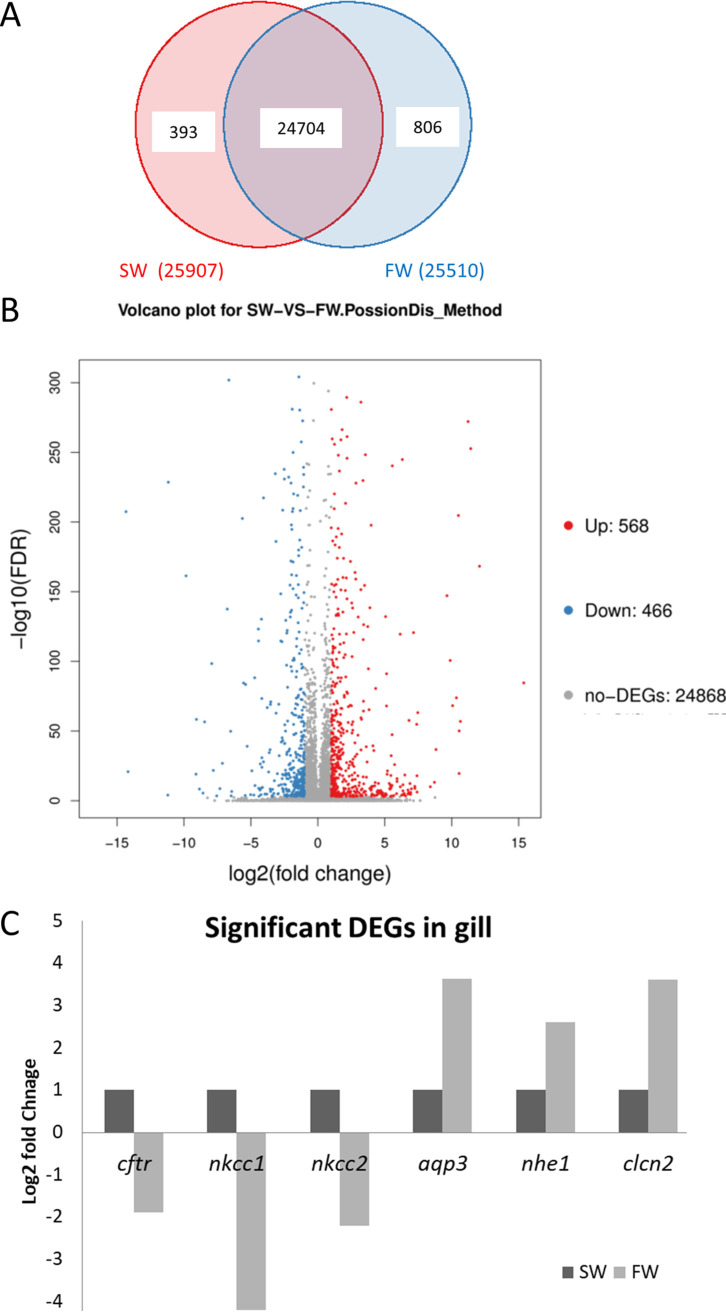
(A) Venn diagram showing the expressed gene between the seawater control and fresh water transferred gill samples. A total 25,903 transcripts were identified in which 24,704 transcripts were found in both groups. (B) Volcano plots of DEGs. DEGs identified in SW/FW; 568 upregulated DEGs and 466 downregulated DEGs were identified in the SW/FW group. Red points indicate the upregulated genes, while blue represents the downregulated genes. Nonsignificant transcripts were marked gray. (C) Significant DEGs in FW gill. Selected transporters are shown. The *y* axis presents the log_2_ fold change value.

Sequencing libraries were prepared from extracted gills of SW and FW and run in the BGISEQ-500 platform. The general sequencing information about the clean read quality is summarized in Table S1 in the supplemental material. Briefly, 24.96 million reads per sample, with an average mapping ratio with reference genome of 92.52% and 72.59% of the average mapping ratio with genes, were observed. Venn diagrams showed 25,903 identified transcripts, in which 24,704 transcripts were found commonly in both the control seawater (SW) and fresh water transferred (FW) samples (Fig. 1A). The DEGs with significant differences were used for the downstream bioinformatics analysis.

10.1128/msystems.00047-22.1 TABLE S1 The general sequencing information of the RNA-seq. Download Table S1, XLSX file, 0.01 MB. Copyright © 2022 Lai et al. 2022 Lai et al. https://creativecommons.org/licenses/by/4.0/ This content is distributed under the terms of the Creative Commons Attribution 4.0 International license.

### DEG and enriched KEGG in freshwater transferred gill.

**FIG 2:**
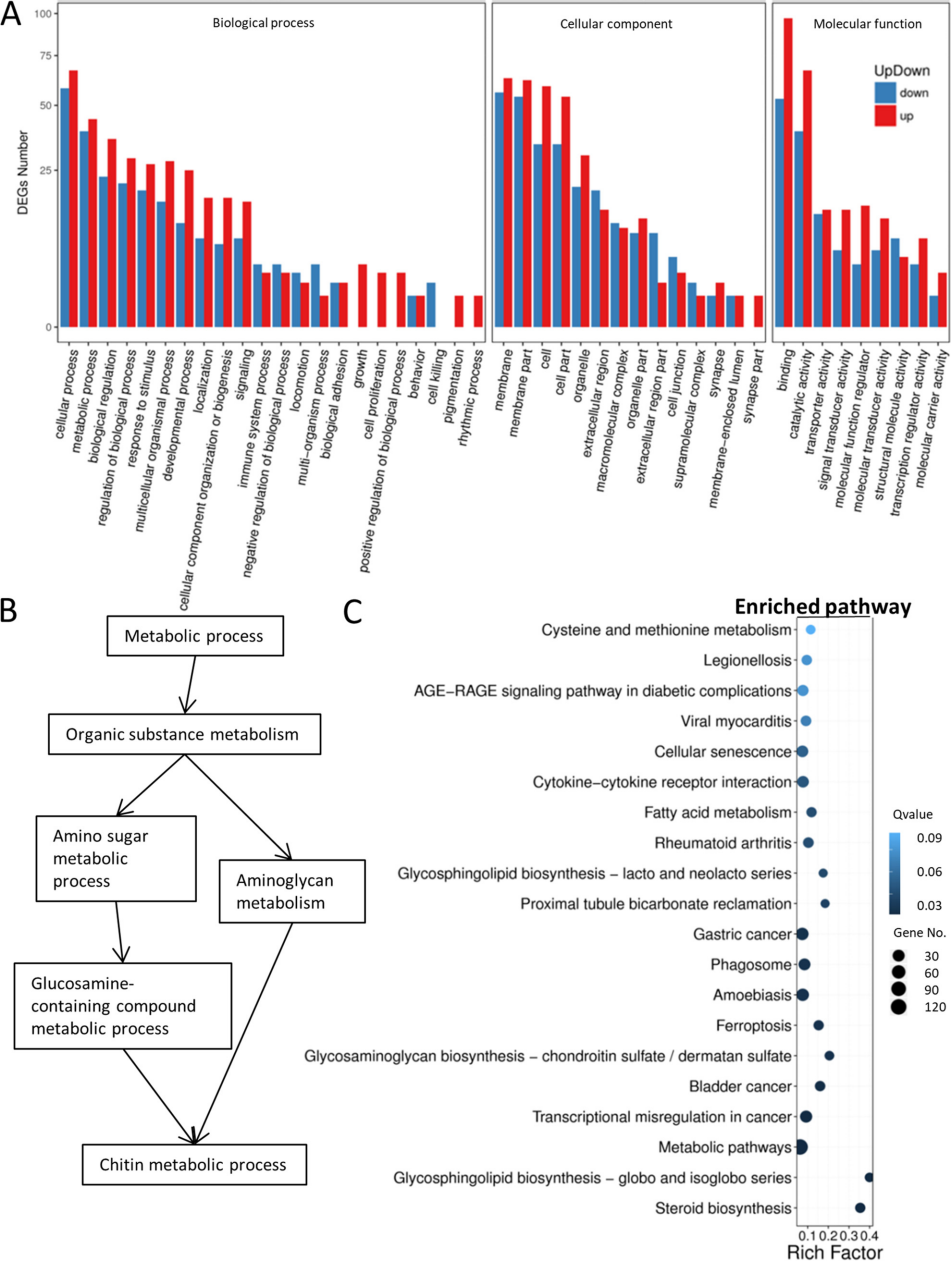
(A) GO classification of DEGs between groups. GO terms identified in SW/FW group. *y* axis shows the number of DEGs, and the *x* axis indicates the GO terms that were classified into biological process, cellular component, and molecular function. Red bars indicate the upregulated genes, while blue indicates the downregulated genes. (B) Directed acyclic graph (DAG) of the selected enriched BP terms. Chitin metabolic process was significantly enriched after fresh water transfer via amino sugar metabolism and aminoglycan metabolism. (C) Pathway functional enrichment of DEGs in SW/FW group. *x* axis represents the enrichment factor, while *y* axis indicates the enriched pathways. The color indicates the *q* value: the blue color represents the lower value, while the white color indicates the higher value. In addition, the point size indicates the number of DEGs. The greater the rich factor, the more significant the enrichment. Glycosaminoglycan and steroid biosynthesis were found to be enriched in the SW/FW group.

After the seawater-to-fresh-water transfer, 1,034 DEGs (up, 568; down, 466) were identified (Fig. 1B). The selected well-known seawater or fresh water transporters’ mRNA expression levels are shown in Fig. 1C. For example, seawater transporters, such as *cftr*, *nkcc1*, and *nkcc2* (5, 25), were suppressed after the fresh water transfer. On the other hand, the fresh water transporters like *aqp3*, *nhe1*, and *clcn2* (5, 26, 27) were upregulated. The full list of the DEGs is in Table S2. All the DEGs then underwent the GO analysis. The top enriched GO terms in biological process (BP), cellular component (CC), and molecular functions (MF) are shown in Fig. 2A. For BP, metabolic process, response to stimulus, and biological adhesion were found. Osmotic stress stimulated the cell and triggered the modifications of the adhesion proteins for osmoregulatory mechanisms. Cell migration is a suggested phenotype during the osmoregulatory progresses (28). Cells have to modulate cell–cell adhesion and gain motility to move (29, 30). Epithelial-mesenchymal transition (EMT) is a critical process to change cell adhesion and polarity (31–33). Such progress involves the increase of mesenchymal markers but decrease of epithelial markers (34, 35) that could explain the reason for identifying similar gene enrichment counts on biological adhesion in this study. For the CC and MF, membrane and transporter activity were ranked as one of the top enrichment terms that reflected the importance of the modification of gill ion transporters that were located in the cell membrane. The full list of the enriched GO terms is shown in Table S3. When we presented the BP category data as a directed acyclic graph (DAG), the chitin metabolic process was significantly enriched via amino sugar metabolism and aminoglycan metabolism (Fig. 2B). The details of the DAG are in Fig. S1.

10.1128/msystems.00047-22.2 TABLE S2 List of DEGs after fresh water transfer. Download Table S2, XLSX file, 0.2 MB. Copyright © 2022 Lai et al. 2022 Lai et al. https://creativecommons.org/licenses/by/4.0/ This content is distributed under the terms of the Creative Commons Attribution 4.0 International license.

10.1128/msystems.00047-22.3 TABLE S3 List of enriched GO terms after fresh water transfer. Download Table S3, XLSX file, 0.1 MB. Copyright © 2022 Lai et al. 2022 Lai et al. https://creativecommons.org/licenses/by/4.0/ This content is distributed under the terms of the Creative Commons Attribution 4.0 International license.

10.1128/msystems.00047-22.4 FIG S1 Directed acyclic graph (DAG) of the enriched BP category after fresh water transfer. Download FIG S1, PDF file, 0.03 MB. Copyright © 2022 Lai et al. 2022 Lai et al. https://creativecommons.org/licenses/by/4.0/ This content is distributed under the terms of the Creative Commons Attribution 4.0 International license.

KEGG pathway analysis was performed to show the functional enrichment from the DEGs. The full lists of the enriched terms (level 2) are shown in Table S4. Pathways such as fatty acid metabolism, glycosphingolipid biosynthesis, glycosaminoglycan biosynthesis, and steroid biosynthesis were enriched (Fig. 2C). The KEGG-DEG relationship network was drafted to have a more detailed understanding of the whole regulatory system in both groups. The network clearly showed that the metabolic pathway was linked with the mentioned glycosphingolipid and steroid biosynthesis. Readers can refer to the PDF file (Fig. S2).

10.1128/msystems.00047-22.5 TABLE S4 List of enriched KEGG pathways after fresh water transfer. Download Table S4, XLSX file, 0.05 MB. Copyright © 2022 Lai et al. 2022 Lai et al. https://creativecommons.org/licenses/by/4.0/ This content is distributed under the terms of the Creative Commons Attribution 4.0 International license.

10.1128/msystems.00047-22.6 FIG S2 The KEGG-DEG network after fresh water transfer. Download FIG S2, PDF file, 1.7 MB. Copyright © 2022 Lai et al. 2022 Lai et al. https://creativecommons.org/licenses/by/4.0/ This content is distributed under the terms of the Creative Commons Attribution 4.0 International license.

### Hypotonic stress alters the gill microbial diversity in marine medaka.

**FIG 3:**
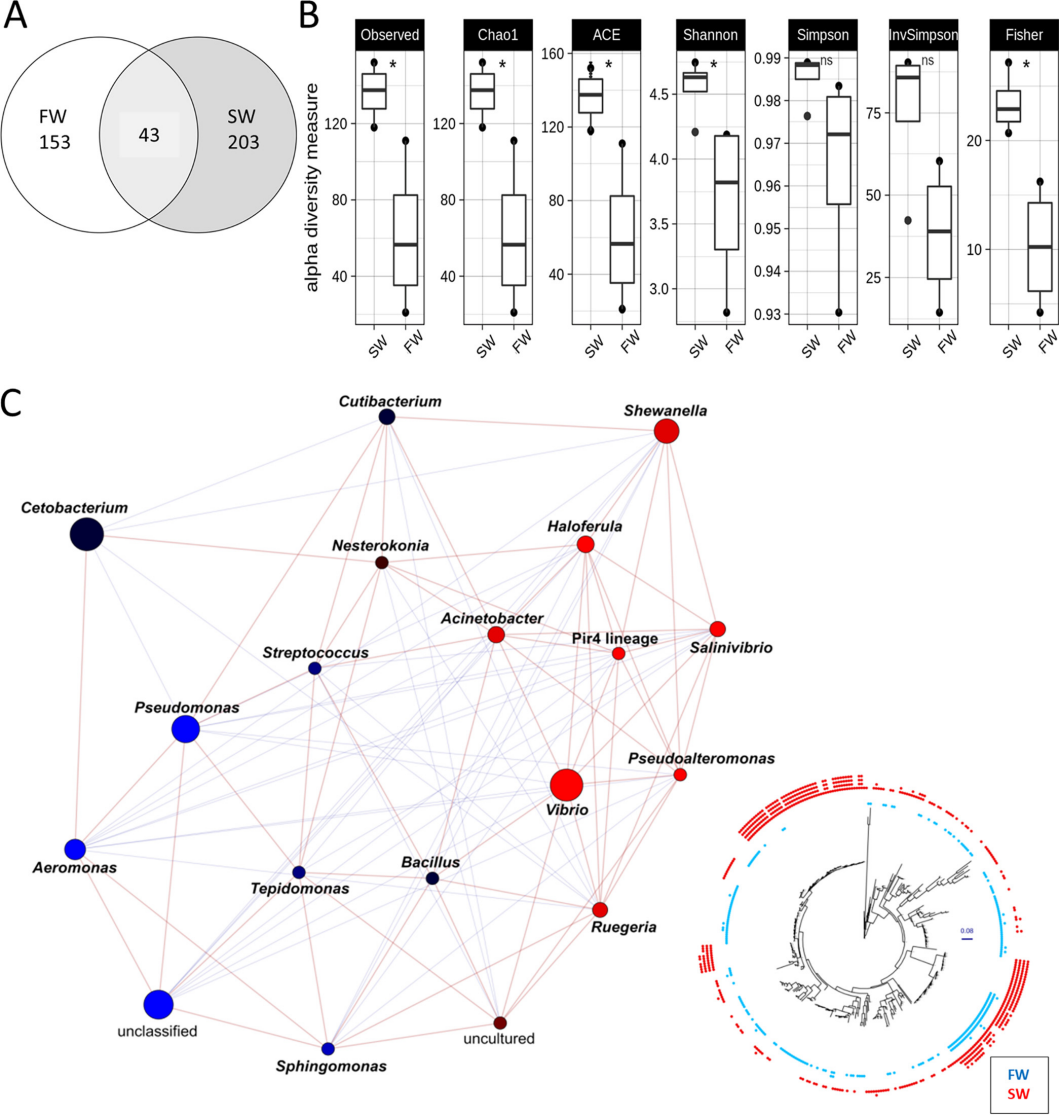
(A) Venn diagram of the ASV detected in the gill of SW control group and the FW group. Different colors represent different groups; the intersection represents the set of ASV commonly present in the counterpart groups. Of the 399 ASV recognized (246 in SW, 196 in FW), 43 were found in common in SW and FW. (B) Various alpha diversity measurements of the two groups. Results indicated that the transfer of fish from SW to FW could lead to significant reduction of gill microbial diversity. (C) The network relationship among the two groups was obtained from Calypso analysis. *Vibrio* was mainly found in SW (red spot), while Pseudomonas was in FW (blue spot). *Cetobacterium* was found in both SW and FW. Tree diagram at the right bottom shows that the FW (blue spot) shares some microbiota with SW (red spot).

The 16S rRNA gene sequencing in gill of marine medaka was performed. A total of 399 sequences (246 in SW, 196 in FW) were identified by the amplicon sequence variant (ASV) method. Among them, 43 sequences showed overlap between SW and FW (Fig. 3A). Alpha diversity was used to analyze the complexity of species diversity in the medaka fish gill (36). Various indexes are shown in Fig. 3B. For example, the Shannon index reflects species diversity of the community, which is affected by both species richness and evenness. Our results showed that FW could lead to a significant reduction of gill microbial diversity compared to SW (Fig. 3B). Moreover, the calypso analysis was performed to obtain a higher-level analysis of microbial community composition (37). The network relation is shown in Fig. 3C. The control SW is shown in red, while the FW data are shown in blue. The result demonstrated that the microbiota communities were changed after the transfer, in which *Vibrio* was spotted in SW and Pseudomonas in FW. *Cetobacterium* was found in both SW and FW. Phylogenetic tree diagram showed that certain microbiotas in FW overlapped with SW (Fig. 3C, right bottom).

### Osmotic stress triggers changes in the fish gill microbial taxonomic composition.

**FIG 4:**
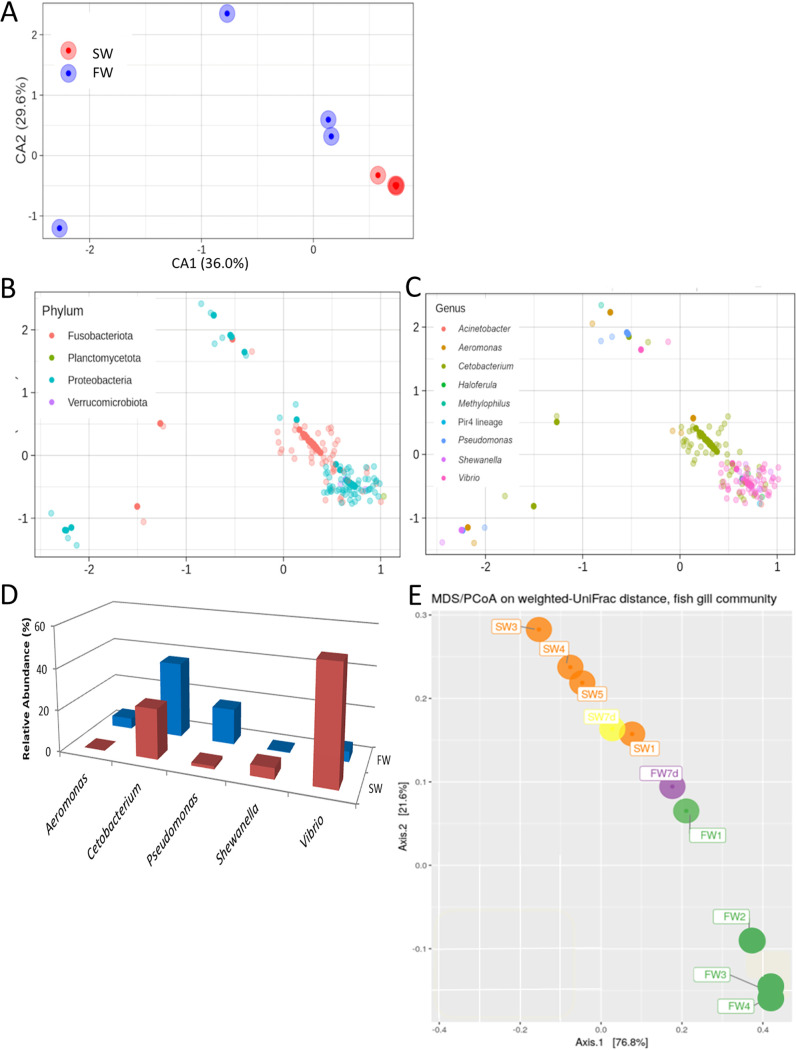
(A) Canonical correspondence analysis (CCA) was used to show the microbiota communities under the two conditions. Distinct compositions were found in the control marine medaka (SW, red spot) and progressive-transfer medaka (FW, blue spot). (B) The microbiota community distribution at phylum level. *Proteobacteria* was mainly found in the samples. (C) The microbiota community distribution at genus level. *Vibrio* (pink spot) was mainly found in SW, while Pseudomonas (blue spot) was in FW. *Cetobacterium* (light green spot) was found in both SW and FW. (D) The relative abundance of selected gill bacteria at genus level. *Vibrio* was highly present in the SW gill. When the fish was transferred to fresh water (FW), *Vibrio* was reduced and Pseudomonas was increased. (E) PCoA of rearing water and gill samples. The SW (orange) and FW (green) gill microbiota were located apart from the seawater (yellow) and the fresh water (purple).

**FIG 5:**
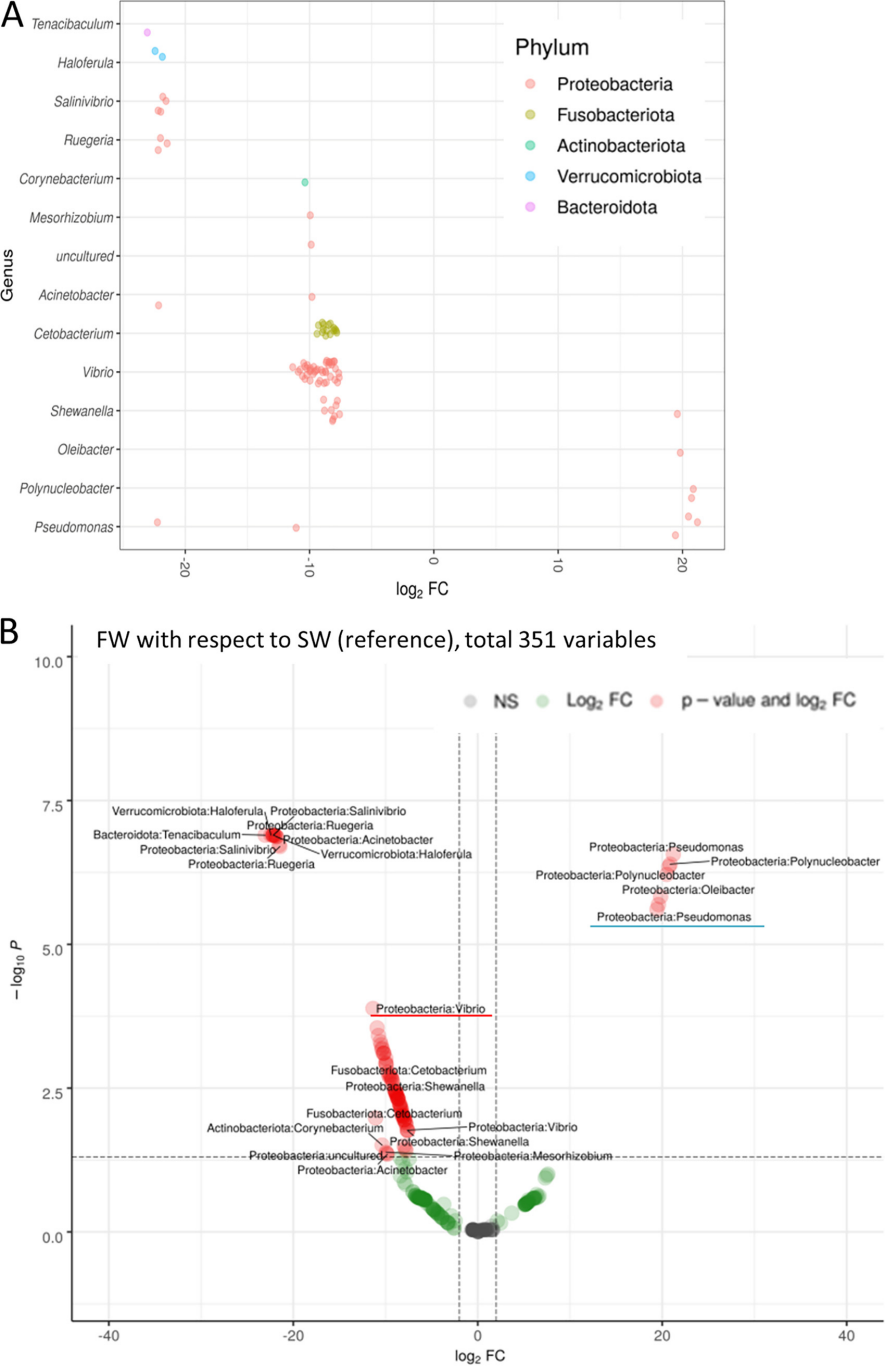

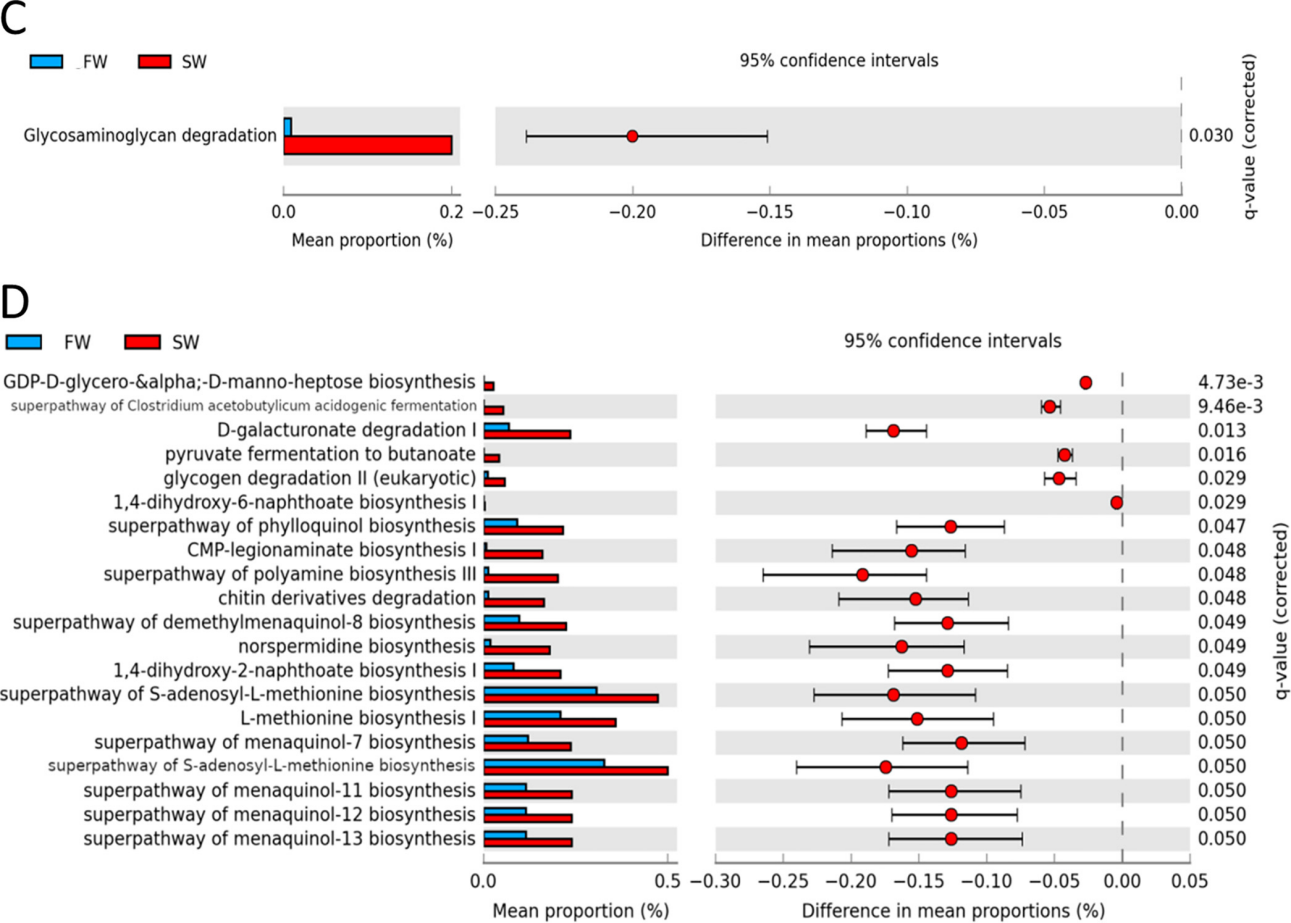
(A) Comparison of microbiota at genus level between the gill of SW control group and the FW group. *y* axis shows the genus, and the *x* axis represents the log_2_ fold value. *Vibrio* was mainly found in SW, while Pseudomonas was dominant in FW gill. (B) Volcano plot of the microbiota. Red indicates the significant changes in abundances, while green refers to changes without statistical significance. SW was used as the reference. *Vibrio* was reduced (red underline) after the fresh water transfer, while Pseudomonas (green underline) increased its abundance. (C) KEGG bioinformatics analysis on the gill microbiota. Only the glycosaminoglycan degradation was enriched significantly in SW group. (D) MetaCyc analysis showed the 20 enriched pathways with significant changes. The bar plot shows mean proportions of differential MetaCyc pathways. The difference in proportions between the groups is shown with 95% confidence intervals. Methionine biosynthesis and chitin derivative degradation were identified that may play roles with the gill cell in osmoregulation.

Distinct diversity patterns between the control marine medaka (SW, red spot), and the fresh water transferred medaka (FW, blue spot) can be seen after the canonical correspondence analysis (CCA) (Fig. 4A). At the phylum level, the control marine medaka contained *Proteobacteria* and *Fusobacteriota*. After the fresh water transfer, these two bacteria could still be identified in the samples (Fig. 4B). At the genus level, *Vibrio* (pink spot) was the dominant bacteria in the control seawater gill, and the Pseudomonas (blue spot) was found mainly in the FW gill. *Cetobacterium* (light green spot) could be found in both SW and FW (Fig. 4C). Regarding the abundances, *Vibrio* (∼55%) was the dominant bacteria at the genus level in the control seawater gill. When the fish was transferred to fresh water, *Vibrio* was reduced to about 5%, and Pseudomonas was increased to 17% in FW. *Cetobacterium* maintained its abundance at around 24% in SW and 37% in FW (Fig. 4D). The raw data of the samples can be found in Table S5. To understand the origins of rising FW bacteria during the transfer, we collected the rearing water samples that underwent the sequencing. The principal coordinate analysis (PCoA) result indicated that the SW gill microbiota (orange) and FW gill (green) were located apart from the seawater (yellow) and the fresh water (purple) (Fig. 4E and Fig. S3). Such result suggested that the changes of external aquatic microbiota composition were not the major factor contributing to the shift of gill bacteria. Such a result matched the notion that the new external aquatic microbiotas were not able to complete with the well-established microbes (38). Lastly, volcano plots were further used to show the microbiota composition differences between SW and FW. At genus level, 14 bacteria had significant differences (Fig. 5A). The hypotonic stress resulted in the decrease of *Vibrio* (red underlined) and increase of Pseudomonas (blue underlined) abundances (Fig. 5B). The full list and statistical data can be referred to (Table S6). Lastly, the KEGG and MetaCyc analyses were performed to identify the possible functional differences between microbiota in SW and FW. In the KEGG analysis, only the glycosaminoglycan degradation was identified in the SW/FW group (Fig. 5C). It should be noted that all the enriched pathways were identified in the SW group, which indicated the gill microbiota after hypotonic stress have relatively lower metabolic activities. Pathways such as chitin derivative degradation and methionine biosynthesis were found (Fig. 5D). The full list with static data can be referred to (Table S7).

10.1128/msystems.00047-22.7 TABLE S5 Raw data of the abundances of microbiota at each level. Download Table S5, XLSX file, 0.2 MB. Copyright © 2022 Lai et al. 2022 Lai et al. https://creativecommons.org/licenses/by/4.0/ This content is distributed under the terms of the Creative Commons Attribution 4.0 International license.

10.1128/msystems.00047-22.8 FIG S3 Bar chart showing the relative abundance of microbiota of water samples and gill samples at genus level. Download FIG S3, PDF file, 0.3 MB. Copyright © 2022 Lai et al. 2022 Lai et al. https://creativecommons.org/licenses/by/4.0/ This content is distributed under the terms of the Creative Commons Attribution 4.0 International license.

10.1128/msystems.00047-22.9 TABLE S6 Raw data of the significant changes between SW/FW. Download Table S6, XLSX file, 0.02 MB. Copyright © 2022 Lai et al. 2022 Lai et al. https://creativecommons.org/licenses/by/4.0/ This content is distributed under the terms of the Creative Commons Attribution 4.0 International license.

10.1128/msystems.00047-22.10 TABLE S7 Raw data of the MetaCyc analysis. Download Table S7, XLSX file, 0.02 MB. Copyright © 2022 Lai et al. 2022 Lai et al. https://creativecommons.org/licenses/by/4.0/ This content is distributed under the terms of the Creative Commons Attribution 4.0 International license.

## DISCUSSION

Fish gill is the first and major tissue that contributes to osmoregulation. Marine medaka has the ability to acclimate in both seawater and fresh water, which indicates its gill undergoes rapid osmoregulatory mechanisms to control ion loss and water gain during hypotonic stress.

### Modification of selected ion transporters/water channel mRNA expression level upon hypotonic stress.

Numerous genes were changed upon the hypotonic stress in gills. Our data clearly showed that the two well-known fresh water channels, chloride channel (*clcn2*) and water channel (*aqp3*), were significantly induced in FW gill. It is known that there are two major gill cells in fishes, which are the pavement cells and the mitochondrion-rich cells (ionocytes) (39). Ionocyctes are believed to play major ion regulatory roles during osmoregulation (40). Fresh water acclimation requires the activation of sodium uptake and acid excretion, chloride uptake, and base secretion (1, 17, 40, 41). Clcn2 belongs to the CLC family of chloride channel/transport proteins. It involves the chloride efflux pathway and takes part in chloride uptake (42), and *clcn2* in eel ionocytes has a higher mRNA expression in fresh water (17, 40). Aqp3 is the water channel and was found to be induced in eel gill after fresh water transfer (25). Moreover, sodium hydrogen exchanger 1 (*nhe1*) was also upregulated in FW, which is involved in sodium ion and proton regulation, known to be highly expressed in fresh water environments (25, 27). In addition, it plays important roles in gaseous exchanges and the acid-base balance in fishes (43–46). On the other hand, seawater transporters like sodium potassium chloride cotransporter, *nkcc1* (*slc12a2*), and *nkcc2* (*slc12a1*) decreased their expression about 2- and 4-fold in FW, respectively. In the seawater environment, *nkcc1* and *cftr* were highly expressed in the seawater-type ionocytes (1, 17, 40, 41, 47). In eel gill, *nkcc1* was found upregulated after seawater transfer (25, 27), while *nkcc2* was suggested to play a transition role in hyperosmotic transfer in red drum (48). Furthermore, *cftr* decreased its mRNA expression in FW and matched other publications on fishes (5, 14, 25). Collectively, our findings in whole gill matched those of other publications on fishes and confirmed the reliability of our sequencing data.

### Enriched hormonal and metabolic pathways upon hypotonic stress.

Steroid biosynthesis and glycosphingolipid biosynthesis were highly enriched after fresh water transfer, indicating their possible chronic roles in fresh water environments. A study in Pacific white shrimp also identified these two pathways upon chronic low-salinity stress (49). Steroid hormones are involved in various cellular functions, such as control metabolism, inflammation, immune functions, and salt and water balance (50). For example, season-dependent changes in immune status and activities of immune cells in fishes were suggested to correlate with changes in the levels of circulating sex hormones (51, 52). Enrichment of steroid hormone biosynthesis pathways during osmoregulation was found in other aquatic organisms, such as cetaceans and white shrimp (49, 53). Moreover, it has been suggested that the salinity tolerance capacities were related to differential expression in immune response genes (54). Various transcriptomics studies further demonstrated the immune-related genes are altered in fresh water environments (55, 56).

On the other hand, glycerophospholipids are glycerol-based phospholipids that are the main component of biological membranes (57). They play roles in protecting the cell surface by maintaining the stability of the plasma membrane via modifying the plasma membrane lipid bilayer (58, 59). To maintain and modify the membrane structure, it is suggested that extra energy is needed, and the identified enriched fatty acid biosynthesis could provide such energy for osmoregulation compensatory processes (60, 61).

### Relationship between fish and bacteria.

Fish have unique and direct interaction with the surrounding water. They contact microorganisms throughout their lifetime, a relationship that may be beneficial or pathogenic. The gut microbes have been extensively studied, and researchers identified the host-microbial relationship that contributed to nutritional provisioning, metabolic homeostasis, and immune response (62, 63). Diet composition and osmotic stress were the factors that alter the gut microbiota composition (24, 64, 65). On the other hand, studies of gill bacteria were extremely limited and were mainly focused on the pathological infection issues (66, 67). Currently, a study of gills of reef fish has suggested that the gill microbiome composition differed significantly from that of the gut for both adults and juveniles across 15 teleost fish families (68). However, to our knowledge, there is no study on the effects of osmotic stress in gill microbiota. The adaptive microbial community shifts concomitant with the host habitat change may contribute the necessary physiological changes required for host survival (22).

In the first part of the study, we suggested that the enriched fatty acid biosynthesis provides extra energy for osmoregulation in gill cell. A study showed that the enhanced sphingolipid synthesis could improve osmotic tolerance in bacteria (69). Thus, it is reasonable to link the host-bacterium interaction in the fish gill. In the second part of the study, we aimed to identify the effects of hypotonic stress on gill microbiota communities and further determine the common enriched pathways that were shared by the gill cells and its bacteria.

### The core microbial habitat in fish gill and its osmosensing mechanism.

Studies in fish guts have identified that *Proteobacteria* is a major phylum in numerous species and were found to compose 90% of the gut microbiota (22, 70–72). *Vibrio* was found to be the most dominant reported microbiota in marine fish, while Pseudomonas and *Cetobacterium* were found in the fresh water fishes (24, 63, 73, 74). Here, our result showed that the dominant microbiota in the external tissue (gill) was similar to that of the internal organ (gut). *Vibrio*, as the dominant bacterium in the gill of marine medaka, was suggested to have an evolutionary association with marine fish (75). It has been shown to produce hydrolytic enzymes for breaking down dietary carbohydrates and lipids (76, 77). On the other hand, *Cetobacterium* isolates under the phylum *Fusobacteria* were increased after fresh water transfer (FW). It has been detected in different freshwater fish guts (71, 73) and was shown to produce vitamin B_12_ (78, 79).

Osmoregulatory changes for acclimation can be divided into two major stages, which are the adaptive period with changes in osmotic parameters and the chronic regulatory period for achieving homeostasis (80). During the adaptive phase, changes in external osmolality could induce water fluxes across the cytoplasmic membrane that lead to the modification of intracellular environments such as cellular hydration, molecular crowding, magnitude of turgor, and cellular integrity (81). For the prolonged osmoregulatory mechanism, metabolic pathway activation plays a major role. The enriched methionine metabolism in this study is linked to folate cycle, and the one-carbon metabolism of the histone-like nucleoid structuring protein is a key regulator in sensing the change in osmolality (82).

### Shared enriched chitin-related pathways in gill DEGs and gill bacteria.

The study tried to identify the shared pathways that were enriched in both gill cells and gill bacteria. The shared glycosaminoglycan and chitin metabolism (Fig. 2B and C and 5C and D) may provide hints for the bacterium-host relationship in gill during osmotic stress. Chitin is a cellulose-like biopolymer that is mainly found in marine invertebrates, insects, fungi, and yeasts (83). Chitinase-like proteins have been found to be involved in immune response (84). A previous study in crab demonstrated that the gills could participate in chitin degradation and may prevent pathogen infection (85). However, its function in fish gill is not known. On the other hand, glycosaminoglycan and aminoglycan metabolic processes were shown to be enriched in various cancers (86, 87), similar to chitin, their roles in fish gill osmoregulation are poorly understood. It is not possible to confirm the osmoregulatory functions of these pathways by the current bioinformatics analysis. Nevertheless, the study here suggested chitin and glycoaminoglycan metabolism are important in fish gill osmoregulatory events. Further investigation on this group of genes in the future might discover their new roles in fish osmoregulation.

### Conclusions.

This is the first study using the transcriptome and 16S rRNA gene sequencing to report the hypotonic responsive genes in gill cells and the compositions of gill microbiota in marine medaka. The overlapping glycosaminoglycan- and chitin-related pathways suggest the host-bacterium interaction in fish gill during osmotic stress.

## MATERIALS AND METHODS

### Fish maintenance and experimental setup.

Six-month-old marine medaka (*O. melastigma*) was maintained in seawater at 26°C. Thirty fish were transferred to 50% seawater for 7 days, followed by fresh water for another 7 days before gill isolation for RNA sequencing and 16S rRNA gene sequencing. The transfer experiment was based on our previously published protocol (24). Seawater-to-seawater transfer was performed in another 30 fish as the control group (SW). Fish were kept in one tank for each condition after the transfer. Gill samples were extracted after the transfer. All experimental protocols were approved by the ethics committee of Kyushu University, Japan (A19-165-1).

### Library construction and Illumina RNA-seq.

Total RNA from seawater and fresh water transferred gills was extracted for transcriptome sequencing (RNA-seq) by TRIzol reagent (Life Technologies, CA, USA). Five gills (one side) were pooled as one biological sample, with each group containing four replicates for library construction. The sequencing method was based on our previously published protocol (5). Briefly, RNA concentrations were measured using a Qubit RNA assay kit on a Qubit 2.0 fluorometer (Life Technologies, CA, USA); 300 ng total RNA with an RNA integrity number of >8 was used for library construction (Agilent Technologies, CA, USA). The Agilent 2100 Bioanalyzer system was used for qualification. cDNA libraries were prepared using the TruSeq stranded mRNA LT sample prep kit (Illumina, San Diego, USA) per the protocol. Index codes were ligated to identify the individual samples. mRNA was purified from total RNA using poly(T) oligonucleotide-attached magnetic beads (Illumina, San Diego, USA) before fragmentation. cDNAs were then synthesized by using random oligonucleotides and SuperScript II with DNA polymerase I and RNase H treatment. Overhangs were blunted by treatment with exonuclease/polymerase followed by 3′-end adenylation and ligation to Illumina PE adaptor oligonucleotides. DNA fragments with adaptor molecules on both ends were enriched by using the Illumina PCR primer cocktail for 15 PCR cycles. Products were purified using the AMPure XP system and quantified by the Agilent Bioanalyzer 2100 system and then sequenced by the BGISEQ-500 platform. Sequence reads were filtered by SOAPunke software (v1.5.2) to remove reads with adaptors, >0.1% unknown bases (N), and low-quality reads (i.e., percentage of bases with quality of less than 20 is greater than 50% in a read).

Sequencing reads were mapped to the reference genome using Bowtie2 (v2.2.5) (88) with the parameter settings “-q –phred64 –sensitive –dpad 0 –gbar 99999999 –mp 1,1 –np 1 –score-min L,0,-0.1 -p 16 -k 200,” and then we calculated gene expression level with the RSME software package (89) with default settings to estimate gene and isoform expression levels from RNA-seq data. The DEGs were detected by PossionDis with a fold change of ≥2.0 and false discovery rate (FDR) of ≤0.001 (90). GO and KEGG analysis were performed by using phyper, a function of R.

### 16S rRNA metagenomic sequencing.

Five gill samples (the other side of the same fish) were pooled as one sample, with each group containing four replicates for metagenomic 16S rRNA gene sequencing. Rearing seawater and fresh water were collected at the end of the experiment. Bacterial DNA was extracted by using the DNeasy blood and tissue kit (Qiagen, Hilden, Germany). Bacterial genomic DNA was then collected and quantified using the Qubit dsDNA HS assay kit (Life Technologies, Carlsbad, CA, USA) as previously described (91).

A 30-ng sample of genomic DNA was subjected to amplicon PCR reaction to amplify the DNA fragment flanking the V3 and V4 regions of the 16S rRNA gene as previously described (91). Primer sequences were the following: forward primer, 5′-TCG TCG GCA GCG TCA GAT GTG TAT AAG AGA CAG CCT ACG GGN GGC WGC AG-3′; reverse primer, 5′-GTC TCG TGG GCT CGG AGA TGT GTA TAA GAG ACA GGA CTA CHV GGG TAT CTA ATC C-3′. PCR was performed as initial denaturation at 95°C for 3 min, followed by 25 cycles of denaturation at 95°C for 30 s, annealing at 55°C for 30 s, elongation at 72°C for 30 s, and a final elongation step at 72°C for 5 min. The 16S V3 and V4 amplicon was purified from free primers and primer-dimer species using Ampure XP beads (Agencourt Bioscience, Beverly, MA, USA). The library was quantified using real-time quantitative PCR and quality checked using an Agilent 2100 bioanalyzer instrument (EvaGreen; Santa Clara, CA, USA). The normalized library was then sequenced by the BGI sequencer platform.

### Bioinformatics analysis, data processing, and ASV prediction.

Bioinformatics analysis was carried out by following a workflow that includes (i) quality assessment and preprocessing of raw sequence reads with adapter, primer, and base quality trimming, (ii) custom reference database generation, (iii) amplicon sequence variant (ASV) prediction and taxonomy assignment, (iv) statistical analysis, and (v) visualization. Unless stated otherwise, data handling, statistics, and visualization were performed using custom-made Python, Perl, and R scripts.

The quality of raw sequence data was assessed based on the presence of adapters, primers, and low-quality bases. The presence of these in the data was addressed by trimming the bases off from both ends of the reads using either standalone or a combination of FastQC v0.11.8, Cutadapt v2.10, and Trim Galore v0.6.6 while maintaining a minimum Phred quality score of 20 and length of 150 bp for paired-end (PE) reads. A taxonomy classifier based on 341F-805R universal primer (92), which corresponds to the V3-V4 hypervariable region in the 16S small subunit ribosomal DNA, was built from Silva 138.1 SSU NR99 reference database (Silva 138) (93–95) using RESCRIPt plugin in Qiime v2 (Qiime2), 2020.6 release (96). In brief, Silva 138 was processed starting with trimming of low-quality sequences, filtering sequences by length and taxonomy (900, 1,200, and 1,400 bases for archaea, bacteria, and eukaryotes, respectively), dereplicating sequences and taxonomy by lowest common ancestor (LCA), and extracting amplicon-specific region classifier by Qiime2 feature-classifier extract-reads function. Meanwhile, amplicon sequences were processed and analyzed by Qiime2 pipeline involving steps that import, merge, denoise, and classify features from PE reads and associated metadata into a collection of Qiime2 artifact and visualization files. While —p-min-fold-parent-over-abundance was set to 2, no further trimming and truncation of sequences were carried out during denoising. A quality control step was carried out on denoised sequences to address un- or poorly characterized features using Qiime2 “quality-control exclude seqs” module at 97% identity threshold and 95% query alignment, searching against full-length Silva 138 with vsearch. Features classified as chloroplast or mitochondria were excluded from the data set. Phylogenetic analysis was performed on ASV using Qiime2 alignment and phylogeny functions, generating a midpoint-rooted tree. Features represented as a table, sequence, distance matrix, phylogenetic tree, and biom files were exported individually and combined into a phyloseq object using qiime2R for downstream analysis.

### Taxonomic and functional analysis.

Taxonomic profiling was carried using phyloseq package (97) in R 4.0.2. The resulting phylogenetic tree was visualized using the plot_tree function in the phyloseq package and was annotated with ggtree package (98, 99) in R. Significant difference on alpha diversity indices between SW and FW groups was tested using one-way analysis of variance followed by *post hoc* Tukey honestly significant difference at a 95% confidence level. Likewise, significant differentially abundant taxa at various levels between treatments were identified using DESeq2 (100). Inferences derived from DESeq2 were based on Wald test for two groups with a parametric fit for dispersion and Benjamini-Hochberg correction for multiple testing at a 95% confidence level. On the other hand, functional profiling was performed using PICRUSt2 v2.0.0-b with built-in EC/MetaCyc and KEGG/KO databases (101–103). MetaCyc and KEGG pathway abundances were further analyzed using statistical analysis of taxonomic and functional profiles (STAMP) v2.1.3 (104). Statistical significance in pathway enrichments was calculated between treatments based on White’s nonparametric *t* test (two-sided) with bootstrapping at 95% confidence intervals and Storey’s FDR for multiple testing at the 5% significance level. Finally, cooccurrence network analysis at the family and genus levels was done using Calypso v8.84 with default parameters (37).

### Data accessibility.

The sequencing data from this study have been submitted to the NCBI BioProject (https://www.ncbi.nlm.nih.gov/bioproject) under the accession numbers PRJNA702883 and PRJNA588335.

## References

[1] Hwang PP, Lee TH, Lin LY. 2011. Ion regulation in fish gills: recent progress in the cellular and molecular mechanisms. Am J Physiol Regul Integr Comp Physiol 301:R28–R47. doi:10.1152/ajpregu.00047.2011. 21451143

[2] Mizuhira V, Amakawa T, Yamashina S, Shirai N, Utida S. 1970. Electron microscopic studies on the localization of sodium ions and sodium-potassium-activated adenosinetriphosphatase in chloride cells of eel gills. Exp Cell Res 59:346–348. doi:10.1016/0014-4827(70)90613-0. 4244188

[3] Wong CKC, Chan DKO. 2001. Effects of cortisol on chloride cells in the gill epithelium of Japanese eel, Anguilla japonica. J Endocrinol 168:185–192. doi:10.1677/joe.0.1680185. 11139782

[4] Gu J, Li JW, Tse WK, Chan TF, Lai KP, Wong CK. 2015. Transcriptomic responses of corpuscle of Stannius gland of Japanese eels (Anguilla japonica) to changes in water salinity. Sci Rep 5:9836. doi:10.1038/srep09836. 25907828 PMC5386212

[5] Lai KP, Li JW, Gu J, Chan TF, Tse WK, Wong CK. 2015. Transcriptomic analysis reveals specific osmoregulatory adaptive responses in gill mitochondria-rich cells and pavement cells of the Japanese eel. BMC Genomics 16:1072. doi:10.1186/s12864-015-2271-0. 26678671 PMC4683740

[6] Tse WKF, Sun J, Zhang HM, Lai KP, Gu J, Qiu JW, Wong CKC. 2014. iTRAQ-based quantitative proteomic analysis reveals acute hypo-osmotic responsive proteins in the gills of the Japanese eel (Anguilla japonica). J Proteomics 105:133–143. doi:10.1016/j.jprot.2014.01.025. 24503184

[7] Tse WKF, Sun J, Zhang H, Law AYS, Yeung BHY, Chow SC, Qiu J-W, Wong CKC. 2013. Transcriptomic and iTRAQ proteomic approaches reveal novel short-term hyperosmotic stress responsive proteins in the gill of the Japanese eel (Anguilla japonica). J Proteomics 89:81–94. doi:10.1016/j.jprot.2013.05.026. 23735544

[8] Wong MK, Ozaki H, Suzuki Y, Iwasaki W, Takei Y. 2014. Discovery of osmotic sensitive transcription factors in fish intestine via a transcriptomic approach. BMC Genomics 15:1134. doi:10.1186/1471-2164-15-1134. 25520040 PMC4377849

[9] Roberts TR. 1998. Systematic observations on tropical Asian medakas or ricefishes of the genus Oryzias, with descriptions of four new species. Ichthyol Res 45:213–224. doi:10.1007/BF02673919.

[10] Tse WKF, Lai KP, Takei Y. 2011. Medaka osmotic stress transcription factor 1b (Ostf1b/TSC22D3-2) triggers hyperosmotic responses of different ion transporters in medaka gill and human embryonic kidney cells via the JNK signalling pathway. Int J Biochem Cell Biol 43:1764–1775. doi:10.1016/j.biocel.2011.08.013. 21907305

[11] Miyanishi H, Inokuchi M, Nobata S, Kaneko T. 2016. Past seawater experience enhances seawater adaptability in medaka, Oryzias latipes. Zool Lett 2:12. doi:10.1186/s40851-016-0047-2. PMC4908718 27307998

[12] Inoue K, Takei Y. 2002. Diverse adaptability in Oryzias species to high environmental salinity. Zool Sci 19:727–734. doi:10.2108/zsj.19.727. 12149572

[13] Kim HS, Lee BY, Han J, Jeong CB, Hwang DS, Lee MC, Kang HM, Kim DH, Lee D, Kim J, Choi IY, Lee JS. 2018. The genome of the marine medaka Oryzias melastigma. Mol Ecol Resour 18:656–665. doi:10.1111/1755-0998.12769. 29451363

[14] Lai KP, Li JW, Wang SY, Chiu JMY, Tse A, Lau K, Lok S, Au DWT, Tse WKF, Wong CKC, Chan TF, Kong RYC, Wu RSS. 2015. Tissue-specific transcriptome assemblies of the marine medaka Oryzias melastigma and comparative analysis with the freshwater medaka Oryzias latipes. BMC Genomics 16:135. doi:10.1186/s12864-015-1325-7. 25765076 PMC4352242

[15] Avellán-Llaguno RD, Liu X, Liu L, Dong S, Huang Q. 2020. Elevated bioaccumulation of PFAAs in Oryzias melastigma following the increase of salinity is associated with the up-regulated expression of PFAA-binding proteins. Sci Total Environ 725:138336. doi:10.1016/j.scitotenv.2020.138336. 32298882

[16] Wang SY, Lau K, Lai K-P, Zhang J-W, Tse AC-K, Li J-W, Tong Y, Chan T-F, Wong CK-C, Chiu JM-Y, Au DW-T, Wong AS-T, Kong RY-C, Wu RS-S. 2016. Hypoxia causes transgenerational impairments in reproduction of fish. Nat Commun 7:12114. doi:10.1038/ncomms12114. 27373813 PMC4932196

[17] Hsu HH, Lin LY, Tseng YC, Horng JL, Hwang PP. 2014. A new model for fish ion regulation: identification of ionocytes in freshwater- and seawater-acclimated medaka (Oryzias latipes). Cell Tissue Res 357:225–243. doi:10.1007/s00441-014-1883-z. 24842048

[18] Shen WP, Horng JL, Lin LY. 2011. Functional plasticity of mitochondrion-rich cells in the skin of euryhaline medaka larvae (Oryzias latipes) subjected to salinity changes. Am J Physiol Regul Integr Comp Physiol 300:R858–R868. doi:10.1152/ajpregu.00705.2010. 21191003

[19] Ogoshi M, Kato K, Sakamoto T. 2015. Effect of environmental salinity on expression of adrenomedullin genes suggests osmoregulatory activity in the medaka, Oryzias latipes. Zool Lett 1:12. doi:10.1186/s40851-015-0012-5. PMC4657274 26605057

[20] Bossus MC, Madsen SS, Tipsmark CK. 2015. Functional dynamics of claudin expression in Japanese medaka (Oryzias latipes): response to environmental salinity. Comp Biochem Physiol A Mol Integr Physiol 187:74–85. doi:10.1016/j.cbpa.2015.04.017. 25957710

[21] Caporaso JG, Lauber CL, Costello EK, Berg-Lyons D, Gonzalez A, Stombaugh J, Knights D, Gajer P, Ravel J, Fierer N, Gordon JI, Knight R. 2011. Moving pictures of the human microbiome. Genome Biol 12:R50. doi:10.1186/gb-2011-12-5-r50. 21624126 PMC3271711

[22] Llewellyn MS, McGinnity P, Dionne M, Letourneau J, Thonier F, Carvalho GR, Creer S, Derome N. 2016. The biogeography of the Atlantic salmon (Salmo salar) gut microbiome. ISME J 10:1280–1284. doi:10.1038/ismej.2015.189. 26517698 PMC5029221

[23] Austin B. 2006. The bacterial microflora of fish, revised. ScientificWorldJournal 6:931–945. doi:10.1100/tsw.2006.181. 16906326 PMC5917212

[24] Lai KP, Lin X, Tam N, Ho JCH, Wong MK, Gu J, Chan TF, Tse WKF. 2020. Osmotic stress induces gut microbiota community shift in fish. Environ Microbiol 22:3784–3802. doi:10.1111/1462-2920.15150. 32618094

[25] Tse WKF, Au DWT, Wong CKC. 2006. Characterization of ion channel and transporter mRNA expressions in isolated gill chloride and pavement cells of seawater acclimating eels. Biochem Biophys Res Commun 346:1181–1190. doi:10.1016/j.bbrc.2006.06.028. 16793006

[26] Tse WKF, Wong CKC. 2011. nbce1 and H+-ATPase mRNA expression are stimulated in the mitochondria-rich cells of freshwater-acclimating Japanese eels (Anguilla japonica). Can J Zool 89:348–355. doi:10.1139/z11-009.

[27] Tse WKF, Au DWT, Wong CKC. 2007. Effect of osmotic shrinkage and hormones on the expression of Na+/H+ exchanger-1, Na+/K+/2Cl(-) cotransporter and Na+/K+-ATPase in gill pavement cells of freshwater adapted Japanese eel, Anguilla japonica. J Exp Biol 210:2113–2120. doi:10.1242/jeb.004101. 17562884

[28] Lai KP, Law AYS, Lau MCC, Takei Y, Tse WKF, Wong CKC. 2013. Osmotic stress transcription factor 1b (Ostf1b) promotes migration properties with the modulation of epithelial mesenchymal transition (EMT) phenotype in human embryonic kidney cell. Int J Biochem Cell Biol 45:1921–1926. doi:10.1016/j.biocel.2013.05.023. 23732111

[29] Hartsock A, Nelson WJ. 2008. Adherens and tight junctions: structure, function and connections to the actin cytoskeleton. Biochim Biophys Acta 1778:660–669. doi:10.1016/j.bbamem.2007.07.012. 17854762 PMC2682436

[30] Le Bras GF, Taubenslag KJ, Andl CD. 2012. The regulation of cell-cell adhesion during epithelial-mesenchymal transition, motility and tumor progression. Cell Adh Migr 6:365–373. doi:10.4161/cam.21326. 22796940 PMC3478259

[31] Yang J, Weinberg RA. 2008. Epithelial-mesenchymal transition: at the crossroads of development and tumor metastasis. Dev Cell 14:818–829. doi:10.1016/j.devcel.2008.05.009. 18539112

[32] Fidler IJ. 2003. Timeline–the pathogenesis of cancer metastasis: the “seed and soil” hypothesis revisited. Nat Rev Cancer 3:453–458. doi:10.1038/nrc1098. 12778135

[33] Lim J, Thiery JP. 2012. Epithelial-mesenchymal transitions: insights from development. Development 139:3471–3486. doi:10.1242/dev.071209. 22949611

[34] Hay ED. 1995. An overview of epithelio-mesenchymal transformation. Acta Anat (Basel) 154:8–20. doi:10.1159/000147748. 8714286

[35] Leggett SE, Hruska AM, Guo M, Wong IY. 2021. The epithelial-mesenchymal transition and the cytoskeleton in bioengineered systems. Cell Commun Signal 19:32. doi:10.1186/s12964-021-00713-2. 33691719 PMC7945251

[36] Schloss PD, Westcott SL, Ryabin T, Hall JR, Hartmann M, Hollister EB, Lesniewski RA, Oakley BB, Parks DH, Robinson CJ, Sahl JW, Stres B, Thallinger GG, Van Horn DJ, Weber CF. 2009. Introducing mothur: open-source, platform-independent, community-supported software for describing and comparing microbial communities. Appl Environ Microbiol 75:7537–7541. doi:10.1128/AEM.01541-09. 19801464 PMC2786419

[37] Zakrzewski M, Proietti C, Ellis JJ, Hasan S, Brion M-J, Berger B, Krause L. 2017. Calypso: a user-friendly web-server for mining and visualizing microbiome-environment interactions. Bioinformatics 33:782–783. doi:10.1093/bioinformatics/btw725. 28025202 PMC5408814

[38] Zarkasi KZ, Taylor RS, Abell GCJ, Tamplin ML, Glencross BD, Bowman JP. 2016. Atlantic salmon (Salmo salar L.) gastrointestinal microbial community dynamics in relation to digesta properties and diet. Microb Ecol 71:589–603. doi:10.1007/s00248-015-0728-y. 26780099

[39] Evans DH, Piermarini PM, Choe KP. 2005. The multifunctional fish gill: dominant site of gas exchange, osmoregulation, acid-base regulation, and excretion of nitrogenous waste. Physiol Rev 85:97–177. doi:10.1152/physrev.00050.2003. 15618479

[40] Hiroi J, McCormick SD. 2012. New insights into gill ionocyte and ion transporter function in euryhaline and diadromous fish. Respir Physiol Neurobiol 184:257–268. doi:10.1016/j.resp.2012.07.019. 22850177

[41] Hwang PP, Lee TH. 2007. New insights into fish ion regulation and mitochondrion-rich cells. Comp Biochem Physiol A Mol Integr Physiol 148:479–497. doi:10.1016/j.cbpa.2007.06.416. 17689996

[42] Schiffhauer ES, Vij N, Kovbasnjuk O, Kang PW, Walker D, Lee S, Zeitlin PL. 2013. Dual activation of CFTR and CLCN2 by lubiprostone in murine nasal epithelia. Am J Physiol Lung Cell Mol Physiol 304:L324–L331. doi:10.1152/ajplung.00277.2012. 23316067 PMC3602739

[43] Haswell MS, Randall DJ, Perry SF. 1980. Fish gill carbonic anhydrase: acid-base regulation or salt transport? Am J Physiol 238:R240–R245. doi:10.1152/ajpregu.1980.238.3.R240. 6768312

[44] Randall DJ, Brauner C. 1998. Interactions between ion and gas transfer in freshwater teleost fish. Comp Biochem Physiol A Mol Integr Physiol 119:3–8. doi:10.1016/s1095-6433(97)00412-1. 11253798

[45] Rahim SM, Delaunoy JP, Laurent P. 1988. Identification and immunocytochemical localization of two different carbonic anhydrase isoenzymes in teleostean fish erythrocytes and gill epithelia. Histochemistry 89:451–459. doi:10.1007/BF00492602. 3139588

[46] Gilmour KM. 2012. New insights into the many functions of carbonic anhydrase in fish gills. Respir Physiol Neurobiol 184:223–230. doi:10.1016/j.resp.2012.06.001. 22706265

[47] Dymowska AK, Hwang PP, Goss GG. 2012. Structure and function of ionocytes in the freshwater fish gill. Respir Physiol Neurobiol 184:282–292. doi:10.1016/j.resp.2012.08.025. 22981968

[48] Esbaugh AJ, Cutler B. 2016. Intestinal Na+, K+, 2Cl- cotransporter 2 plays a crucial role in hyperosmotic transitions of a euryhaline teleost. Physiol Rep 4:e13028. doi:10.14814/phy2.13028. 27881573 PMC5358003

[49] Chen K, Li E, Li T, Xu C, Wang X, Lin H, Qin JG, Chen L. 2015. Transcriptome and molecular pathway analysis of the hepatopancreas in the Pacific white shrimp Litopenaeus vannamei under chronic low-salinity stress. PLoS One 10:e0131503. doi:10.1371/journal.pone.0131503. 26147449 PMC4492601

[50] Frye CA. 2009. Steroids, reproductive endocrine function, and affect. A review. Minerva Ginecol 61:541–562. 19942840

[51] Szwejser E, Verburg-van Kemenade BM, Maciuszek M, Chadzinska M. 2017. Estrogen-dependent seasonal adaptations in the immune response of fish. Horm Behav 88:15–24. doi:10.1016/j.yhbeh.2016.10.007. 27760301

[52] Chaves-Pozo E, García-Ayala A, Cabas I. 2018. Effects of sex steroids on fish leukocytes. Biology 7:9. doi:10.3390/biology7010009. PMC5872035 29315244

[53] Birukawa N, Ando H, Goto M, Kanda N, Pastene LA, Nakatsuji H, Hata H, Urano A. 2005. Plasma and urine levels of electrolytes, urea and steroid hormones involved in osmoregulation of cetaceans. Zool Sci 22:1245–1257. doi:10.2108/zsj.22.1245. 16357473

[54] Norman JD, Ferguson MM, Danzmann RG. 2014. Transcriptomics of salinity tolerance capacity in Arctic charr (Salvelinus alpinus): a comparison of gene expression profiles between divergent QTL genotypes. Physiol Genomics 46:123–137. doi:10.1152/physiolgenomics.00105.2013. 24368751 PMC3921346

[55] Gu J, Dai S, Liu H, Cao Q, Yin S, Lai KP, Tse WKF, Wong CKC, Shi H. 2018. Identification of immune-related genes in gill cells of Japanese eels (Anguilla japonica) in adaptation to water salinity changes. Fish Shellfish Immunol 73:288–296. doi:10.1016/j.fsi.2017.12.026. 29269288

[56] Lin G, Zheng M, Gao D, Li S, Fang W, Huang J, Xie J, Liu J, Liu Y, Li Z, Lu J. 2020. Hypoosmotic stress induced tissue-specific immune responses of yellowfin seabream (Acanthopagrus latus) revealed by transcriptomic analysis. Fish Shellfish Immunol 99:473–482. doi:10.1016/j.fsi.2020.02.028. 32070785

[57] Tocher DR. 1995. Chapter 6. Glycerophospholipid metabolism, p 119–157. *In* Hochachka PW, Mommsen TP (ed), Biochemistry and molecular biology of fishes, vol 4. Elsevier, New York, NY.

[58] Bartke N, Hannun YA. 2009. Bioactive sphingolipids: metabolism and function. J Lipid Res 50(Suppl):S91–S96. doi:10.1194/jlr.R800080-JLR200. 19017611 PMC2674734

[59] Brown DA, London E. 2000. Structure and function of sphingolipid- and cholesterol-rich membrane rafts. J Biol Chem 275:17221–17224. doi:10.1074/jbc.R000005200. 10770957

[60] Luvizotto-Santos R, Lee J, Branco Z, Bianchini A, Nery L. 2003. Lipids as energy source during salinity acclimation in the euryhaline crab Chasmagnathus granulata dana, 1851 (crustacea-grapsidae). J Exp Zool 295A:200–205. doi:10.1002/jez.a.10219. 12541304

[61] Lemos D, Phan VN, Alvarez G. 2001. Growth, oxygen consumption, ammonia-N excretion, biochemical composition and energy content of Farfantepenaeus paulensis Pérez-Farfante (Crustacea, Decapoda, Penaeidae) early postlarvae in different salinities. J Exp Mar Biol Ecol 261:55–74. doi:10.1016/s0022-0981(01)00260-x. 11438105

[62] Gomez GD, Balcazar JL. 2008. A review on the interactions between gut microbiota and innate immunity of fish. FEMS Immunol Med Microbiol 52:145–154. doi:10.1111/j.1574-695X.2007.00343.x. 18081845

[63] Sullam KE, Essinger SD, Lozupone CA, O’Connor MP, Rosen GL, Knight ROB, Kilham SS, Russell JA. 2012. Environmental and ecological factors that shape the gut bacterial communities of fish: a meta-analysis. Mol Ecol 21:3363–3378. doi:10.1111/j.1365-294X.2012.05552.x. 22486918 PMC3882143

[64] Liu H, Guo X, Gooneratne R, Lai R, Zeng C, Zhan F, Wang W. 2016. The gut microbiome and degradation enzyme activity of wild freshwater fishes influenced by their trophic levels. Sci Rep 6:24340. doi:10.1038/srep24340. 27072196 PMC4829839

[65] Duan Y, Wang Y, Liu Q, Dong H, Li H, Xiong D, Zhang J. 2019. Changes in the intestine microbial, digestion and immunity of Litopenaeus vannamei in response to dietary resistant starch. Sci Rep 9:6464. doi:10.1038/s41598-019-42939-8. 31015554 PMC6478684

[66] Marcos-López M, Rodger HD. 2020. Amoebic gill disease and host response in Atlantic salmon (Salmo salar L.): a review. Parasite Immunol 42:e12766. doi:10.1111/pim.12766. 32564378

[67] Mitchell SO, Rodger HD. 2011. A review of infectious gill disease in marine salmonid fish. J Fish Dis 34:411–432. doi:10.1111/j.1365-2761.2011.01251.x. 21401646

[68] Pratte ZA, Besson M, Hollman RD, Stewart FJ. 2018. The gills of reef fish support a distinct microbiome influenced by host-specific factors. Appl Environ Microbiol 84:e00063-18. doi:10.1128/AEM.00063-18. 29453266 PMC5930318

[69] Zhu G, Yin N, Luo Q, Liu J, Chen X, Liu L, Wu J. 2020. Enhancement of sphingolipid synthesis improves osmotic tolerance of Saccharomyces cerevisiae. Appl Environ Microbiol 86:e02911-19. doi:10.1128/AEM.02911-19. 32033944 PMC7117927

[70] Givens CE, Ransom B, Bano N, Hollibaugh JT. 2015. Comparison of the gut microbiomes of 12 bony fish and 3 shark species. Mar Ecol Prog Ser 518:209–223. doi:10.3354/meps11034.

[71] Di Maiuta N, Schwarzentruber P, Schenker M, Schoelkopf J. 2013. Microbial population dynamics in the faeces of wood-eating loricariid catfishes. Lett Appl Microbiol 56:401–407. doi:10.1111/lam.12061. 23461380

[72] Ghanbari M, Kneifel W, Domig KJ. 2015. A new view of the fish gut microbiome: advances from next-generation sequencing. Aquaculture 448:464–475. doi:10.1016/j.aquaculture.2015.06.033.

[73] Silva FC, Nicoli JR, Zambonino-Infante JL, Kaushik S, Gatesoupe FJ. 2011. Influence of the diet on the microbial diversity of faecal and gastrointestinal contents in gilthead sea bream (Sparus aurata) and intestinal contents in goldfish (Carassius auratus). FEMS Microbiol Ecol 78:285–296. doi:10.1111/j.1574-6941.2011.01155.x. 21692817

[74] Egerton S, Culloty S, Whooley J, Stanton C, Ross RP. 2018. The gut microbiota of marine fish. Front Microbiol 9:873. doi:10.3389/fmicb.2018.00873. 29780377 PMC5946678

[75] Schmidt VT, Smith KF, Melvin DW, Amaral-Zettler LA. 2015. Community assembly of a euryhaline fish microbiome during salinity acclimation. Mol Ecol 24:2537–2550. doi:10.1111/mec.13177. 25819646

[76] Ray AK, Ghosh K, Ringø E. 2012. Enzyme-producing bacteria isolated from fish gut: a review. Aquaculture Nutr 18:465–492. doi:10.1111/j.1365-2095.2012.00943.x.

[77] Gatesoupe F-J, Infante J-LZ, Cahu C, Quazuguel P. 1997. Early weaning of seabass larvae, Dicentrarchus labrax: the effect on microbiota, with particular attention to iron supply and exoenzymes. Aquaculture 158:117–127. doi:10.1016/S0044-8486(97)00179-8.

[78] Tsuchiya C, Sakata T, Sugita H. 2008. Novel ecological niche of Cetobacterium somerae, an anaerobic bacterium in the intestinal tracts of freshwater fish. Lett Appl Microbiol 46:43–48. doi:10.1111/j.1472-765X.2007.02258.x. 17944860

[79] Sugita H, Shibuya K, Shimooka H, Deguchi Y. 1996. Antibacterial abilities of intestinal bacteria in freshwater cultured fish. Aquaculture 145:195–203. doi:10.1016/S0044-8486(96)01319-1.

[80] Soengas JL, Sangiao-Alvarellos S, Laiz-Carrión R, Mancera JM. 2019. Energy metabolism and osmotic acclimation in teleost fish, p 284–314. *In* Fish osmoregulation. CRC Press, Boca Raton, FL.

[81] Bremer E, Krämer R. 2019. Responses of microorganisms to osmotic stress. Annu Rev Microbiol 73:313–334. doi:10.1146/annurev-micro-020518-115504. 31180805

[82] Landgraf JR, Levinthal M, Danchin A. 1994. The role of H-NS in one carbon metabolism. Biochimie 76:1063–1070. doi:10.1016/0300-9084(94)90031-0. 7748928

[83] Sivanesan I, Gopal J, Muthu M, Shin J, Oh JW. 2021. Reviewing chitin/chitosan nanofibers and associated nanocomposites and their attained medical milestones. Polymers 13:2330. doi:10.3390/polym13142330. 34301087 PMC8309474

[84] van Eijk M, Voorn-Brouwer T, Scheij SS, Verhoeven AJ, Boot RG, Aerts JMFG. 2010. Curdlan-mediated regulation of human phagocyte-specific chitotriosidase. FEBS Lett 584:3165–3169. doi:10.1016/j.febslet.2010.06.001. 20541547

[85] Li J, Sun J, Dong X, Geng X, Qiu G. 2019. Transcriptomic analysis of gills provides insights into the molecular basis of molting in Chinese mitten crab (Eriocheir sinensis). PeerJ 7:e7182. doi:10.7717/peerj.7182. 31293829 PMC6601604

[86] Tsai HT, Huang CS, Tu CC, Liu CY, Huang CJ, Ho YS, Tu SH, Tseng LM, Huang CC. 2020. Multi-gene signature of microcalcification and risk prediction among Taiwanese breast cancer. Sci Rep 10:18276. doi:10.1038/s41598-020-74982-1. 33106505 PMC7588423

[87] Ucakturk E, Akman O, Sun X, Baydar DE, Dolgun A, Zhang F, Linhardt RJ. 2016. Changes in composition and sulfation patterns of glycoaminoglycans in renal cell carcinoma. Glycoconj J 33:103–112. doi:10.1007/s10719-015-9643-1. 26662466

[88] Langmead B, Salzberg SL. 2012. Fast gapped-read alignment with Bowtie 2. Nat Methods 9:357–359. doi:10.1038/nmeth.1923. 22388286 PMC3322381

[89] Li B, Dewey CN. 2011. RSEM: accurate transcript quantification from RNA-seq data with or without a reference genome. BMC Bioinformatics 12:323. doi:10.1186/1471-2105-12-323. 21816040 PMC3163565

[90] Audic S, Claverie JM. 1997. The significance of digital gene expression profiles. Genome Res 7:986–995. doi:10.1101/gr.7.10.986. 9331369

[91] Klindworth A, Pruesse E, Schweer T, Peplies J, Quast C, Horn M, Glockner FO. 2013. Evaluation of general 16S ribosomal RNA gene PCR primers for classical and next-generation sequencing-based diversity studies. Nucleic Acids Res 41:e1. doi:10.1093/nar/gks808. 22933715 PMC3592464

[92] Herlemann DP, Labrenz M, Jürgens K, Bertilsson S, Waniek JJ, Andersson AF. 2011. Transitions in bacterial communities along the 2000 km salinity gradient of the Baltic Sea. ISME J 5:1571–1579. doi:10.1038/ismej.2011.41. 21472016 PMC3176514

[93] Glöckner FO, Yilmaz P, Quast C, Gerken J, Beccati A, Ciuprina A, Bruns G, Yarza P, Peplies J, Westram R, Ludwig W. 2017. 25 years of serving the community with ribosomal RNA gene reference databases and tools. J Biotechnol 261:169–176. doi:10.1016/j.jbiotec.2017.06.1198. 28648396

[94] Quast C, Pruesse E, Yilmaz P, Gerken J, Schweer T, Yarza P, Peplies J, Glöckner FO. 2013. The SILVA ribosomal RNA gene database project: improved data processing and web-based tools. Nucleic Acids Res 41:D590–D596. doi:10.1093/nar/gks1219. 23193283 PMC3531112

[95] Yilmaz P, Parfrey LW, Yarza P, Gerken J, Pruesse E, Quast C, Schweer T, Peplies J, Ludwig W, Glöckner FO. 2014. The SILVA and “all-species living tree project (LTP)” taxonomic frameworks. Nucleic Acids Res 42:D643–D648. doi:10.1093/nar/gkt1209. 24293649 PMC3965112

[96] Bolyen E, Rideout JR, Dillon MR, Bokulich NA, Abnet CC, Al-Ghalith GA, Alexander H, Alm EJ, Arumugam M, Asnicar F, Bai Y, Bisanz JE, Bittinger K, Brejnrod A, Brislawn CJ, Brown CT, Callahan BJ, Caraballo-Rodríguez AM, Chase J, Cope EK, Da Silva R, Diener C, Dorrestein PC, Douglas GM, Durall DM, Duvallet C, Edwardson CF, Ernst M, Estaki M, Fouquier J, Gauglitz JM, Gibbons SM, Gibson DL, Gonzalez A, Gorlick K, Guo J, Hillmann B, Holmes S, Holste H, Huttenhower C, Huttley GA, Janssen S, Jarmusch AK, Jiang L, Kaehler BD, Kang KB, Keefe CR, Keim P, Kelley ST, Knights D, et al. 2019. Reproducible, interactive, scalable and extensible microbiome data science using QIIME 2. Nat Biotechnol 37:852–857. doi:10.1038/s41587-019-0209-9. 31341288 PMC7015180

[97] McMurdie PJ, Holmes S. 2013. phyloseq: an R package for reproducible interactive analysis and graphics of microbiome census data. PLoS One 8:e61217. doi:10.1371/journal.pone.0061217. 23630581 PMC3632530

[98] Yu G. 2020. Using ggtree to visualize data on tree-like structures. Curr Protoc Bioinformatics 69:e96. doi:10.1002/cpbi.96. 32162851

[99] Yu G, Lam TT, Zhu H, Guan Y. 2018. Two methods for mapping and visualizing associated data on phylogeny using Ggtree. Mol Biol Evol 35:3041–3043. doi:10.1093/molbev/msy194. 30351396 PMC6278858

[100] Love MI, Huber W, Anders S. 2014. Moderated estimation of fold change and dispersion for RNA-seq data with DESeq2. Genome Biol 15:550. doi:10.1186/s13059-014-0550-8. 25516281 PMC4302049

[101] Czech L, Barbera P, Stamatakis A. 2020. Genesis and Gappa: processing, analyzing and visualizing phylogenetic (placement) data. Bioinformatics 36:3263–3265. doi:10.1093/bioinformatics/btaa070. 32016344 PMC7214027

[102] Barbera P, Kozlov AM, Czech L, Morel B, Darriba D, Flouri T, Stamatakis A. 2019. EPA-ng: massively parallel evolutionary placement of genetic sequences. Syst Biol 68:365–369. doi:10.1093/sysbio/syy054. 30165689 PMC6368480

[103] Douglas GM, Maffei VJ, Zaneveld JR, Yurgel SN, Brown JR, Taylor CM, Huttenhower C, Langille MGI. 2020. PICRUSt2 for prediction of metagenome functions. Nat Biotechnol 38:685–688. doi:10.1038/s41587-020-0548-6. 32483366 PMC7365738

[104] Parks DH, Tyson GW, Hugenholtz P, Beiko RG. 2014. STAMP: statistical analysis of taxonomic and functional profiles. Bioinformatics 30:3123–3124. doi:10.1093/bioinformatics/btu494. 25061070 PMC4609014

