# Supplementary figures and images for: Integrated Omics Approaches Revealed the Osmotic Stress-Responsive Genes and Microbiota in Gill of Marine Medaka
Source: mSystems. 2022 Mar 14;7(2):e00047-22. doi: 10.1128/msystems.00047-22 (PMC9040874; doi:10.1128/msystems.00047-22)

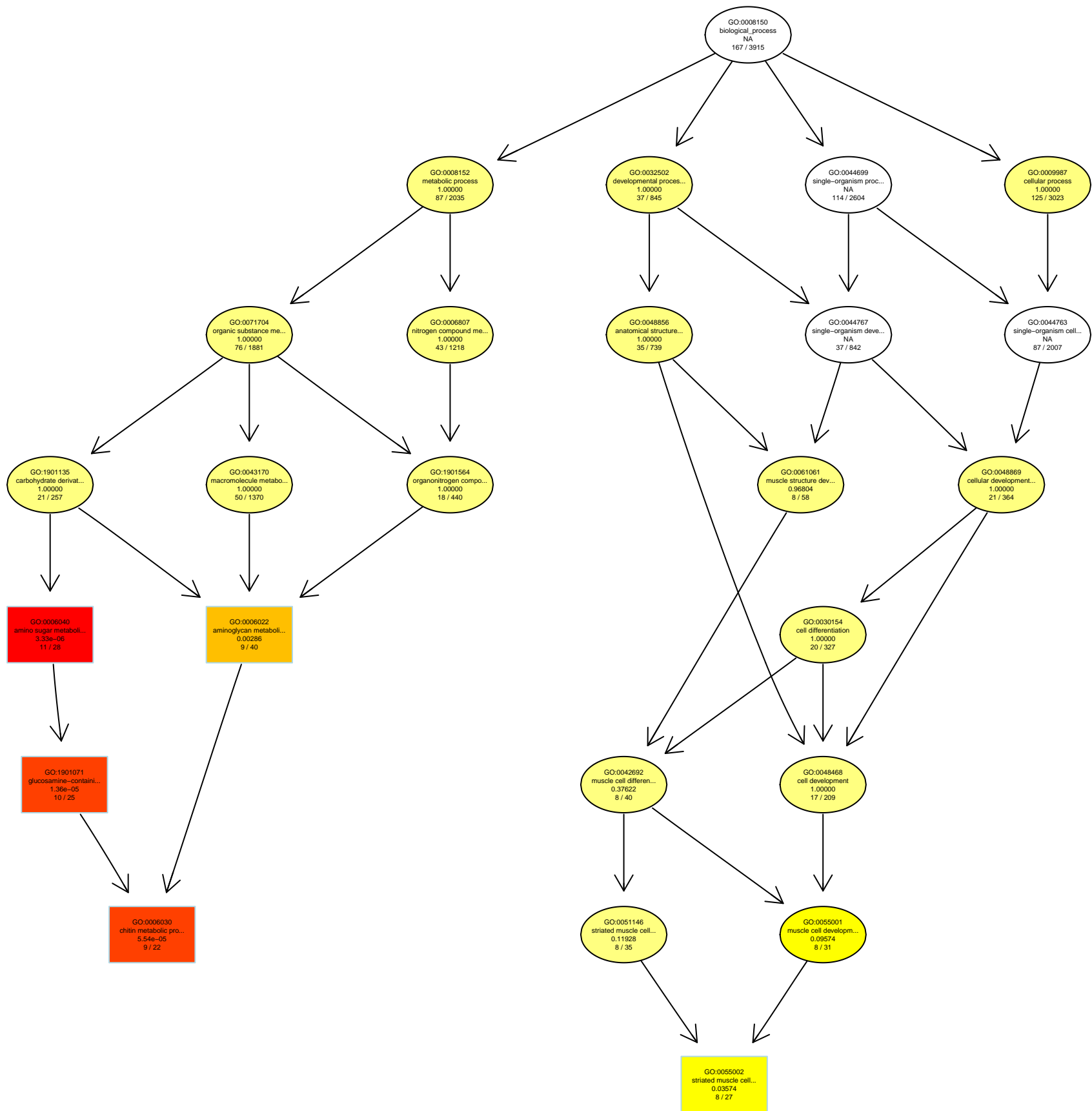

Supplement: FIG S1 [file msystems.00047-22-sf001.pdf]

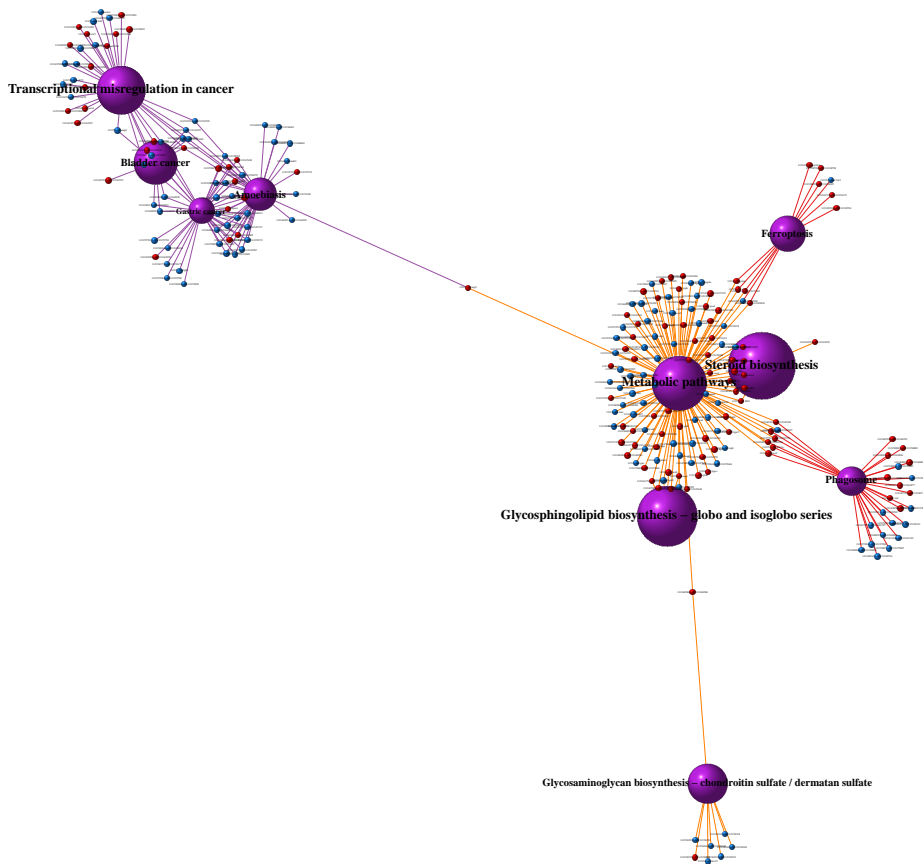

Supplement: FIG S2 [file msystems.00047-22-sf002.pdf]

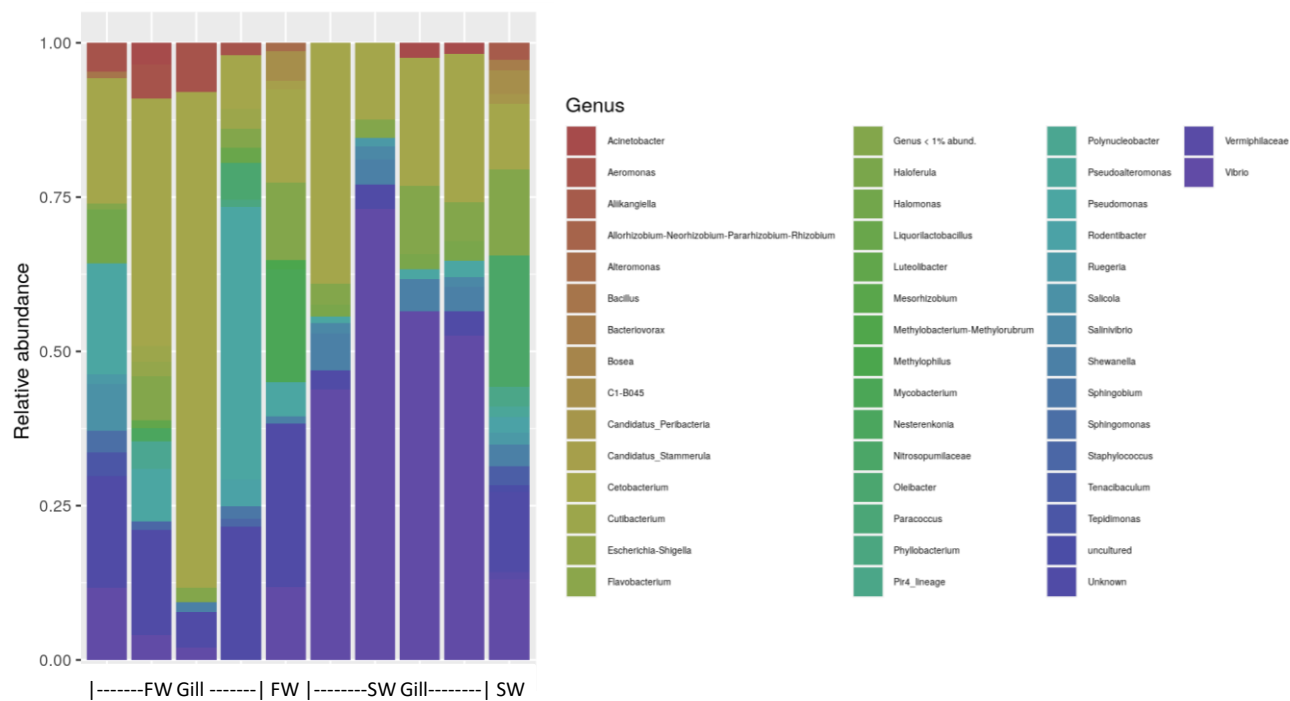

Supplement: FIG S3 [file msystems.00047-22-sf003.pdf]
